# Altered Bile Acid Transport in Liver Disease

**DOI:** 10.3390/biomedicines14051037

**Published:** 2026-05-02

**Authors:** Sarah Cayton, Lindsay C. Czuba

**Affiliations:** Department of Pharmaceutical Sciences, College of Pharmacy, University of Kentucky, Lexington, KY 40536, USA; sarah.cayton@uky.edu

**Keywords:** bile acids, bile acid transporters, liver disease, fibrosis, apical sodium-dependent bile acid transporter (ASBT), organic anion transporting polypeptides (OATPs)

## Abstract

**Background/Objectives:** Bile acids, synthesized from cholesterol in the liver, are amphipathic molecules that play an integral role in lipid digestion and absorption, while also serving as systemic endocrine hormones. They continuously undergo enterohepatic circulation, where they interact with various transporter proteins. Dysregulated bile acid transport is associated with the pathogenesis of liver disease. This review summarizes the key findings relating to bile acid transport expression and activity in the pathogenesis of liver disease. **Methods:** A review of the literature was performed using PubMed and relevant terms including, but not limited to, “bile acid transporters”, “liver disease”, and “bile acid uptake and efflux”. Studies published in peer-reviewed journals relevant to this review were considered and reviewed. **Results:** Within the gut and liver, several key bile acid and xenobiotic transporters within the enterohepatic circulation are dysregulated. The directionality and extent of changes are cell- and disease-specific. Many of the regulatory processes are driven by changes in bile acid signaling, although further work is needed to expand on post-translational modification of bile acid transporters in liver disease. **Conclusions:** Bile acid transporters are dynamically regulated in liver diseases with distinct etiologies. Therefore, restoring BA transporter function represents an actionable therapeutic approach to liver disease.

## 1. Introduction

Liver disease is responsible for 4% of all deaths worldwide [1], and the prevalence of the disease is expected to increase. Therefore, understanding the molecular mechanisms of liver disease may help to understand the underlying etiology and trajectory of pathogenesis, and to elucidate potential drug targets. Bile acids are central players in many forms of liver diseases [2]. They are bioactive end-products of cholesterol catabolism that function as dietary emulsifiers, aiding in the absorption of fat-soluble lipids and vitamins [3]. They also function as hormones, signaling via both G-coupled protein receptors and nuclear receptors to maintain and regulate lipid, glucose and energy homeostasis [3]. Excessive levels of bile acids can also be toxic [4]. Under cholestatic conditions, bile acids exert toxicity to hepatocytes by causing membrane disruption, oxidative stress, and, ultimately, apoptosis [4]. Thus, the body must maintain tight control of local bile acid concentrations to ensure adequate levels are available for physiological functions, while protecting against cytotoxicity. In humans, the total hepatic tissue concentration of bile acids is ~60 nmol/g in healthy tissue and predominantly comprises cholic acid, chenodeoxycholic acid, and deoxycholic acid [5,6]. Total concentrations of bile acids rise to ~120 nmol/g for liver tissue in cirrhosis and >225 nmol/g for liver tissue in cholestasis [6]. Lithocholic acid is considered the most toxic bile acid due to its hydrophobicity. However, in liver disease, the hepatic bile acid metabolome is primarily enriched with cholic acid and chenodeoxycholic acid, and deoxycholic acid and lithocholic acid are largely absent [5].

**Figure 1:**
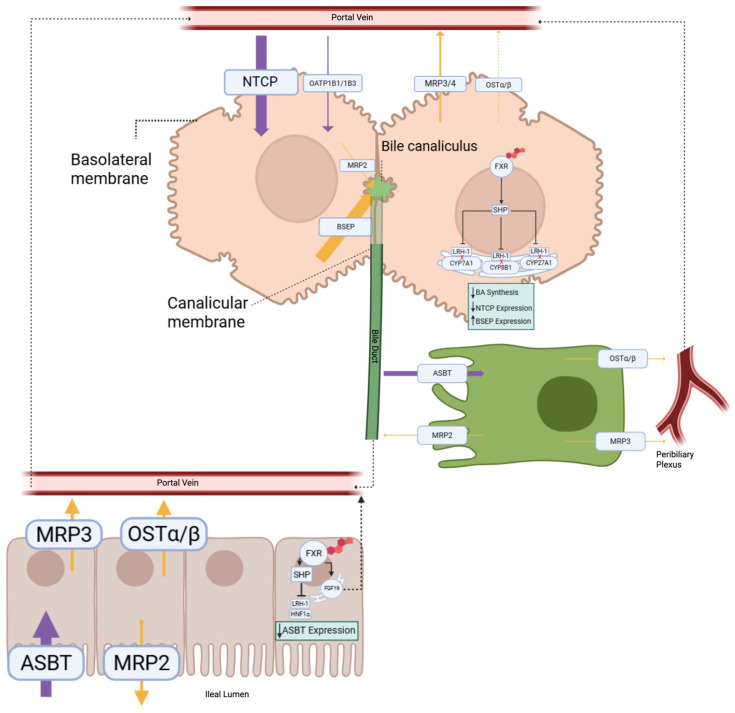
Enterohepatic circulation of bile acids. In the hepatocyte, bile acids are taken up from portal circulation by NTCP and OATP1B1/1B3 across the basolateral membrane. They are exported across the canalicular membrane primarily by BSEP. Postprandially, bile acids will reach the distal ileum after secretion from the gallbladder. They will be reclaimed into the enterocyte by ASBT. After export from the enterocyte by OST alpha/beta, bile acids enter systemic circulation via the portal vein, where they recycle back to the hepatocyte. Arrows represent the direction of movement, with yellow arrows representing efflux and purple arrows representing import.

The maintenance of bile acid homeostasis occurs through a multi-organ process known as enterohepatic circulation (EHC) (Figure 1). The enterohepatic circulation of bile acids begins in the liver with de novo bile acid synthesis and biliary efflux into the bile [7]. Following postprandial secretion from the gallbladder, bile acids are reclaimed by the enterocyte of the distal ileum [4,8]. Upon delivery of bile acids to the basolateral membrane of the ileal enterocytes, bile acids are effluxed from the enterocytes into the lamina propria for subsequent secretion into portal blood [4,9]. Once in portal circulation, bile acids are extracted by the liver [4,10]. This cycle can occur between 4 and 12 times per day, whereby ~95% of the bile acid pool is reclaimed by recycling [4].

The regulation of the enterohepatic circulation of bile acids is critical for maintaining bile acid homeostasis in the body and involves both feed-forward and feedback mechanisms [4]. Specifically, through interactions with the farnesoid X receptor (FXR; *NR1H4*), bile acids regulate their own synthesis and transport [4,11]. Different bile acids have differing affinities for FXR. The strongest affinity bile acid is chenodeoxycholic acid (CDCA), followed by deoxycholic acid (DCA), lithocholic acid (LCA), and then cholic acid (CA) [12]. However, access to FXR and other nuclear targets of bile acid signaling is facilitated in part by their cellular disposition and the activities of bile acid transport proteins.

**Figure 2:**
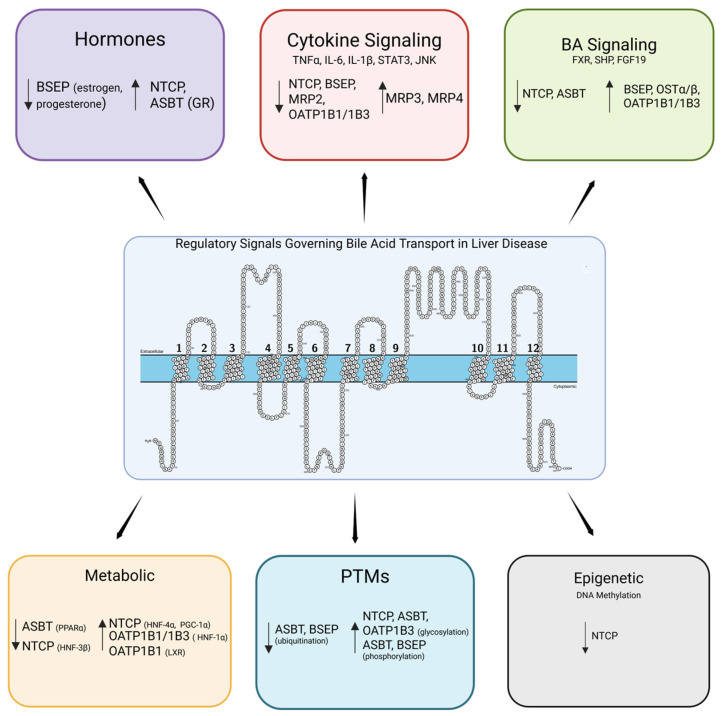
Overview of the regulatory signals seen in liver disease impacting bile acid transport. Key transcriptional, post-translational, and protein interaction pathways that influence bile acid transport are highlighted. Up arrows signify upregulation of a transporter, while down arrows represent downregulation of a transporter. A representative 2D topology model of a bile acid transporter (OATP1B1) is provided, generated using Protter v. 1.0 (UniProt: SO1B1_HUMAN, https://protter.ethz.ch/#up=SO1B1_HUMAN&tm=auto&mc=skyblue&lc=black&tml=numcount&numbers&legend&tex=loopextent{20}&n:signal%20peptide,fc:red,bc:red=UP.SIGNAL&n:disulfide%20bonds,s:box,fc:greenyellow,bc:greenyel-low=UP.DISULFID&n:variants,s:diamond,fc:orange,bc:orange=UP.VARIANT&n:PTMs,s:box,fc:forestgreen,bc:forestgreen=UP.CARBOHYD,UP.MOD_RES&format=svg, accessed 24 April 2026). Together, this represents multiple levels of regulation during disease progression.

This review specifically highlights the impact of bile acid transporters in regulating bile acid homeostasis, providing a mechanistic link between their dysregulation in liver disease etiology and pathogenesis. In addition, this is the first review to integrate post-translational modifications of bile acid transporters across etiologies, drawing critical attention to the dynamic mechanisms regulating transporter functional expression patterns. Many of the key regulatory mechanisms are highlighted in the subsequent sections, along with descriptions of the expression patterns of bile acid transporters (Figure 2) and consequences of their dysregulation within the gut–liver axis (Table 1).

## 2. Key Transporters in the Enterohepatic Circulation of Bile Acids

### 2.1. Apical Sodium-Dependent Bile Acid Transporter (ASBT; SLC10A2)

#### 2.1.1. Cellular Localization and Function

The apical sodium-dependent bile acid transporter (ASBT) is primarily localized on the apical membrane of enterocytes in the distal ileum [43] (Figure 1). The *SLC10A2* gene is located on 13q33 [44]. The protein expression of ASBT is concentrated on the brush-border membrane, where it transports bile acids into the enterocyte along with sodium [45]. In addition, ASBT is also expressed in the proximal tubule cells of the kidney, suggesting a role in renal bile acid handling [45]. The inhibition of the renal ASBT has been explored in cholemic nephropathy, a complication of cholestatic liver disease [46]. Evidence also shows that ASBT is expressed in cholangiocytes [47]. Cholangiocyte expression of ASBT allows for participation in a cholehepatic shunt, where bile acids are recycled between cholangiocytes and hepatocytes through the peribiliary plexus [48]. Overall, the roles of ASBT in the disposition of bile acids in the ileum are well-defined, while it’s roles in the kidney and cholangiocytes remain understudied.

In fasted humans, the median luminal bile acid concentrations in the duodenum and jejunum are ~3 mM [49,50], while postprandially, the median concentrations may be over three times higher [51]. ASBT-mediated uptake of bile acids from the intestine represents the rate-limiting step of enterohepatic circulation and recycling of bile acids to the liver and for which it is highly efficient [43,52]. ASBT shows strong substrate preference for taurine- and glycine-conjugated bile acids, which represent the majority of the postprandial small intestinal bile acid pool [53], and has a higher affinity for hydrophobic bile acids [54]. Despite the increased capability of transporting conjugated bile acids, unconjugated bile acids are still transported by ASBT [54]. Unlike other bile acid transporters, there is little evidence of ASBT-mediated transport on non-bile acid substrates apart from bile acid-conjugated prodrugs.

**Figure 3:**
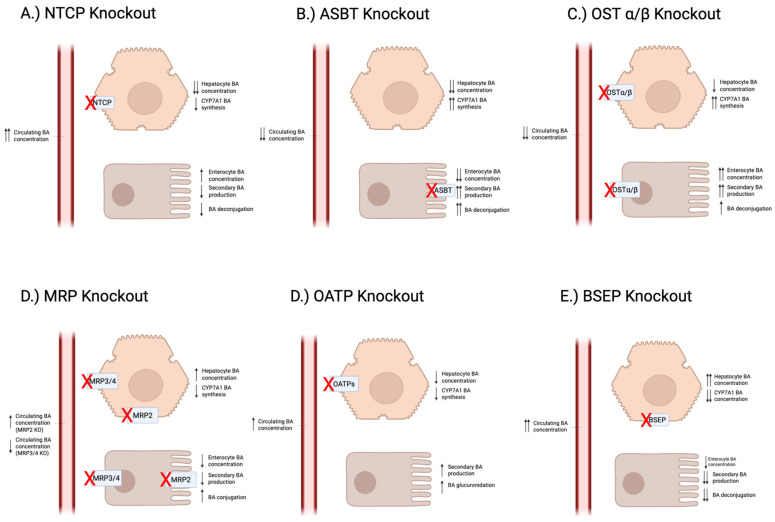
Summarized effects of knockout of bile acid transporters of EHC. Arrows represent the direction of observed metabolic changes.

In support of the overall importance of ASBT in maintaining bile acid homeostasis, bile duct ligation in mice results in ASBT upregulation [55]. Similarly, ASBT knockout mice (Figure 3) exhibit a reduced pool of bile acids, as well as a less hydrophilic bile salt pool, showing the role that ASBT plays in maintaining the bile acid pool [55]. ASBT-knockout mice with bile duct ligation reduced plasma bilirubin and alkaline phosphatase by 57% and 49%, respectively, demonstrating a link between ASBT dysregulation and liver injury [55]. Likewise, humans with a genetic loss of *SLC10A2* exhibit primary bile acid malabsorption (PBAM). PBAM has been associated with increased levels of serum alanine aminotransferase (ALT) and markers of fatty liver disease [56].

#### 2.1.2. Regulation of ASBT Expression and Activity

The expression of ASBT is tightly controlled at the transcriptional level by a network of transcription factors and nuclear receptor signaling pathways. FXR plays an important role in the regulation of ASBT. Bile acids are known to negatively regulate ASBT, which is indirectly regulated through an FXR–bile acid complex [57]. The FXR–bile acid complex activates the small heterodimer partner (SHP), which in turn inhibits RAR:RXR, leading to inhibition of the transcription of *SLC10A2* in response to bile acids [57]. Liver receptor homolog 1 (LRH-1) is activated in mice instead of RAR:RXR, which is a key species difference in the regulation of ASBT [57]. Apart from FXR’s role in the regulation of ASBT, the glucocorticoid receptor (GR) is known to transactivate *SLC10A2* [58]. When bound by a ligand, the GR will translocate to the nucleus and bind glucocorticoid response elements in the *SLC10A2* promoter [58]. This will increase ASBT transcription. PPARα also has a binding site on the *SLC10A2* gene promoter, and it has been shown that PPARα suppresses transcription of *SLC10A2* [59]. PPARα binds the DR1 elements in the *SLC10A2* promoter (nt −1565 to −1577), which will then suppress ASBT transcription, linking fatty acid oxidation to reduced bile acid uptake [59].

Numerous other transcription factors have a direct or indirect link to ASBT expression. For instance, the caudal-type homeobox transcription factors CDX1 and CDX2 also directly bind to and activate the *SLC10A2* promoter, leading to increased intestinal expression of ASBT protein [60]. Another transcription factor, hepatocyte nuclear factor 1 homeobox A (HNF1α), works in tandem with sterol regulatory binding element protein 2 (SREBP2) through an unknown mechanism to promote transcription of *SLC10A2* in the presence of cholesterol [61]. This links cholesterol sensing pathways and bile acid transport regulation, though insight into the molecular basis of this interaction needs further research. Activator protein 1 (AP-1) is a heterodimer transcription factor complex made up of c-Fos, c-Jun or activating transcription factor (ATF) members [62]. In rats, *Asbt* was shown to have two different AP-1 response elements [63,64]. ASBT expression is downregulated by c-Fos and c-Jun through the induction of interleukin 1β (IL-1β) [65]. IL-1β activates JNK signaling, leading to the phosphorylation of c-Jun and increased AP-1 activity, which represses *SLC10A2* transcription in conditions where IL-1β is elevated [65].

After transcription, ASBT is heavily regulated by several post-translational modifications, altering its stability, localization, and activity. ASBT undergoes N-glycosylation at Asn10, producing a mature glycoform of the protein, enhancing protein stability and providing protection from proteolytic degradation [66,67]. Deglycosylation of ASBT reduces transport activity of the protein, though trafficking to the plasma membrane appears to be unaffected [67]. S-acylation of ASBT allows transport activity and bile acid uptake [68]. Furthermore, tyrosine phosphorylation has been shown to be key for membrane stability of ASBT [69]. Inhibition of phosphorylation through the induction of protein tyrosine phosphatases [70] or tyrosine kinase inhibition [69] leads to a reduction in membrane expression, as well as reduced transport capability [69]. This supports the idea that phosphorylation significantly contributes to protein stability and/or location. ASBT palmitoylation and tyrosine phosphorylation appear to be linked, at least in vitro [68]. Similarly, the loss of either of those PTMs promotes degradation by the ubiquitin–proteosome pathways. Collectively, these modifications facilitate the dynamic regulation of ASBT [71] and consequently regulate the intestinal uptake of bile acids and systemic concentrations [72].

### 2.2. Na+/Taurocholate Cotransporting Polypeptide (NTCP; SLC10A1)

#### 2.2.1. Cellular Localization and Function

The Na+/Taurocholate Cotransporting Polypeptide (NTCP) is located on the basolateral membrane of hepatocytes (Figure 1) [73] and is involved in the uptake of bile acids into the liver from nutrient-rich portal blood [44,74]. The portal vein is estimated to have 20–50 µM bile acids compared to ~5 µM in systemic circulation [75]. Within portal circulation, CA is at a concentration of ~8 µM, CDCA is at a concentration of ~6 µM, and DCA is at a concentration of ~6 µM [76]. Unlike intestinal ASBT, NTCP exhibits a wide substrate-specificity and is able to transport bile acids and steroid hormones alike [54]. Despite this wide specificity, NTCP shows preference in transporting conjugated bile acids over unconjugated bile acids [54]. The affinity of cholic acid for NTCP is ~55 µM, while for TCA and GCA it is ~20 µM and ~16 µM, respectively [77]. Apart from its activity as a bile acid transporter, NTCP is involved in the internalization of hepatitis B virus (HBV) into the liver, though this mechanism is not fully understood [78].

#### 2.2.2. Regulation of NTCP Expression and Activity

The *SLC10A1* gene, consisting of five exons, is located on chromosome 14 in humans [79]. High levels of bile acids suppress *SLC10A1* gene expression through activation of FXR in a SHP-mediated pathway [74]. Bile acids bind to FXR in hepatocytes promoting heterodimerization of FXR to the retinoid X receptor (RXR). Heterodimerization of FXR:RXR leads to the induction of the SHP [74]. The SHP lacks a DNA-binding domain but interacts with other transcription factors, often as a corepressor and is critical to the regulation of bile acid homeostasis. For instance, under basal conditions, the transcription factor hepatocyte nuclear factor 4 alpha (HNF-4α) is a direct activator of NTCP transcription [80]. In mice, HNF-4α enhances *SLC10A1* promoter activity in the presence of PGC-1-alpha [80]. Co-transfection of HNF-4a, PGC-1α and mNtcp in hepatoma cells induces *Ntcp* promoter activity by 50% [80]. The transcription factor SHP represses the transactivation activity of HNF-4α by blocking it from binding to its co-activators [81]. Consequently, NTCP expression is downregulated when bile acid concentrations are high in hepatocytes. In contrast to the basal role of HNF-4α in regulating NTCP expression, hepatocyte nuclear factor 3β (HNF-3β) also binds directly to the NTCP promoter at a conserved response element in the 5′-regulatory region, resulting in repression of NTCP gene transcription [82].

Gut–liver endocrine signaling plays an indirect role in the FXR-mediated transcriptional regulation of *SLC10A1*. In mice, the administration of hFGF19 downregulated mRNA levels of NTCP [83]. FGF19 in humans is secreted from the ileal enterocyte upon FXR activation [84]. FGF19 binds to FGFR4/β-Klotho complexes in the hepatocyte, which activates ERK1/2 signaling [85]. This leads to suppression of CYP7A1 mRNA expression, resulting in a reduction in bile acid synthesis, as well a decrease in NTCP expression, reducing bile acid recycling to the liver.

Apart from FXR, other transcriptional signaling pathways influence NTCP expression. *SLC10A1* is also activated by the glucocorticoid receptor (GR). The GR will translocate to the nucleus once the glucocorticoid is bound, where it will then bind glucocorticoid response elements in the *SLC10A1* promoter, enhancing its transcription [74]. This activation is enhanced by PPARα [74]. Elevated signal transducer and activator of transcription 3 (STAT3) levels have been correlated with downregulation of *SLC10A1* mRNA levels [73]. The induction of STAT3 by IL-6 in mice has been shown to repress Slc10a1 mRNA expression, but the mechanism for this downregulation still needs to be defined [86]. Similarly, DNA methylation significantly impacts the promoter activity of *SLC10A1* [73]. Methylation in the CpG islands within the *SLC10A1* promoter reduces the ability of transcription factors to bind to the NTCP promoter, resulting in decreased gene expression [73]. Interleukin 6 (IL-6) induces a dose-dependent reduction in NTCP steady-state mRNA levels [13]. Similarly, IL-1β suppresses NTCP gene expression through a c-Jun N-terminal kinase (JNK)-dependent mechanism. IL-1β-induced activation of JNK leads to phosphorylation of c-Jun, which disrupts RXR transcriptional complexes from interacting with the *SLC10A1* promoter [14]. This mechanism may underly disease-associated changes in hepatocyte regulation of NTCP as well as control NTCP-mediated uptake of its bile acid substrates.

NTCP degradation occurs through multiple post-translational pathways, depending on the state of protein maturation. When protein folding of a newly synthesized NTCP is not carried out correctly, this leads to ER-associated degradation [87]. A properly folded NTCP is exported from the ER and trafficked to the membrane, where it is later internalized and degraded in the lysosome [88]. N-glycosylation of NTCP facilitates localization at the plasma membrane, and when glycosylation sites are mutated, the protein is degraded in the lysosome [88].

### 2.3. Heteromeric Organic Solute Transporter (OSTα: SLC51A; OSTβ: SLC51B)

#### 2.3.1. Cellular Localization and Function

OSTα/β is a heteromeric transporter formed by the dimerization of two subunits encoded by genes located on two different chromosomes. OSTα is encoded at 3q29, while OSTβ is encoded at 15q22 on the human chromosome [89]. While the two proteins function together to act as a transporter, they do have unique structural and functional differences. OSTα is a seven-transmembrane subunit, while OSTβ is 128-amino acid protein with a single transmembrane domain [90]. OSTα is stabilized by the presence of the smaller OSTβ [91]. Co-expression of OSTα and β allows for trafficking of the heterodimer from the endoplasmic reticulum to the plasma membrane, while the individual proteins will not exit the ER [91]. When complexed, they form a functional carrier capable of facilitating transport generally located in the basolateral membrane of epithelial cells [92]. OSTα/β is primarily localized on the basolateral membrane of enterocytes, cholangiocytes, hepatocytes, and renal epithelial cells, allowing efflux of bile acids (Figure 1) [92].

In the intestine, bile acids are effluxed from enterocytes by OSTα/β [93] and prevent ileal toxicity from high bile acid concentrations from the intestinal lumen [94]. In the liver, OSTα/β likely functions as a compensatory transporter for the efflux of bile acids out of the hepatocyte [95]. OSTα/β transports unconjugated and conjugated bile acids alike; however, glycine- and taurine-conjugated bile acids (e.g., TCA) are transported at a much higher capacity than the glycine conjugates (e.g., GCA) [96]. Its substrate preference for bile acids is TCDCA > GCDCA > TCA > GCA for the conjugated, primary bile acids [96]. OSTα/β, in addition to its role in bile acid transport, can transport steroids [97].

#### 2.3.2. Regulation of OSTα/β Expression and Activity

OSTα/β expression is regulated transcriptionally by FXR. FXR heterodimerizes with RXR once bound by a ligand, leading to increased expression of OSTα/β during high bile acid concentrations [98,99]. The FXR:RXR complex binds to the inverted hexameric nucleotide repeat separated by one nucleotide (IR-1) motif in the promoter region of both the *OSTα* and *OSTβ* genes [98]. This allows for the promotion of *SLC51A* and *SLC51B* transcription, leading to formation of the heterodimer.

Loss of intestinal OSTα/β disrupts basolateral efflux of bile acids, leading to enhanced signaling by FGF15/19 [100]. In mice, knockout of Ostα led to a 20-fold increase in FGF15, a downstream gene target of FXR (Figure 2). This ultimately results in a reduction in the total bile acid pool, as well as suppression of Cyp7a1 [100]. This pathway was dependent on the expression of Fxr, as combined Ostα/Fxr knockout mice did not have a reduced bile acid pool [100].

### 2.4. Organic Anion Transporting Polypeptides (OATPs; SLCO Family)

#### 2.4.1. Cellular Localization and Function

The organic anion transporting polypeptides are a family of transporters, of which several play a role in enterohepatic circulation (Figure 1). Within the family, OATP1B1 (*SLCO1B1*), OATP1B3 (*SLCO1B3*), and OATP2B1 (*SLCO2B1*) are basolateral transporters involved in hepatic uptake of bile acids from portal circulation into the hepatocyte [101]. OATP1A2 is also expressed in the liver and intestine [102]. Other OATP family members that may not typically be involved in bile acid handling have been implicated in disease-state bile acid transport. For instance, OATP3A1 mRNA and proteins levels are significantly increased in cholestatic conditions [103].

Knockout models of OATP family members show the importance of these transporters in bile acid handling (Figure 2). In Oatp1b2 knockout mice, the murine equivalent of OATP1B1/1B3, mice have 3- to 45-fold higher serum levels of unconjugated bile acids [104]. In humans, simultaneous deficiency in OATP1B1 and OATP1B3 leads to a condition known as Rotor syndrome, characterized by hyperbilirubinemia [105].

While NTCP is the dominant transporter of conjugated bile acids, OATP1B1 and OATP1B3 also transport sulfated and glucuronidated bile acids, as well as unconjugated bile acids. In addition to their role in bile acid transport, OATP1B1 and OATP1B3 transport bilirubin conjugates, steroid hormones, and several xenobiotics [101]. OATP1B1 plays an important role in the clearance of circulating bile acid metabolites. In patients with Rotor syndrome, 3-glucurondiated bile acids and 3-sulfated bile acids accumulate in systemic circulation [106]. As known substrates of OATPs, sulfated bile acids can be used as an endogenous biomarker to monitor the function of OATPs [107]. OATP2B1 also contributes to hepatic uptake of sulfated bile acids, as well as other organic ions, though bile acids have a lower affinity for OATP2B1 compared to OATP1B1 and OATP1B3 [108].

#### 2.4.2. Regulation of OATP Expression and Activity

Nuclear receptor signaling pathways tightly regulate the expression of OATPs to control bile acid homeostasis. FXR has been shown to directly induce transcription of several OATP transporters. In hepatoma cells, CDCA treatment increased promoter activity of *OATP1B3* in a pathway mediated by an IR-1 response element [109]. When this element was mutated, CDCA was no longer able to induce *OATP1B3* transcription through FXR [109]. OATP1B1 is also regulated by FXR. FXR agonist treatment in primary human hepatocytes and Huh7 cells lead to significant increases in *OATP1B1* mRNA expression and transporter activity [110]. Mutation of IR-1 elements in the promoter led to the loss of FXR-induced transcription [110].

In addition to FXR, LXR has been shown to be an important nuclear receptor in the regulation of OATPs. *OATP1B1* mRNA was again increased in primary human hepatocytes and hepatocyte-derived cell lines when LXR agonists were used to treat the cells [110]. LXR binds to an alternative response element in the *SLCO1B1* promoter, leading to induction of its transcription [110]. In contrast to FXR and LXR, the nuclear receptors PXR and CAR seem to play a minimal role in OATP transcriptional regulation [110]. In monkeys, rifampin, a powerful PXR inducer, led to a less than 2-fold increase in OATP1B1, OATP1B3, and OATP2B1 induction [111].

OATP suppression often occurs in tandem with NTCP downregulation, reflecting a coordinated shutdown of bile acid uptake to limit hepatotoxicity induced by bile acid accumulation. HNF-1α regulates mRNA expression of OATP1B1 through a direct interaction between the transcription factor and promoter region of OATP1B1, inducing its transcription [112]. OATP expression is tightly regulated at the transcriptional level by a number of cytokines. STAT3 activation by IL-6 suppresses HNF-dependent transcriptional control of SLCO genes [86]. IL-6 and TNFα both significantly reduce mRNA expression of *OATP1B1* and *OATP1B3* [16].

In addition to transcriptional regulation, post-translational regulation of OATPs helps further modulate their activity. N-glycosylation influences the trafficking of OATP1B1 protein to the membrane [113]. Asn134 and Asn516 are important N-glycosylation points for OATP1B3, which allow proper expression and transporter activity [114]. When Asn134 is unavailable as an N-glycosylation site, Asn503 is glycosylated instead [114]. Non-glycosylated OATP1B3 remains in the ER as it is no longer able to be properly trafficked to the cell membrane. Further, lysine acetylation at K650, especially concurrent with serine phosphorylation at S659/S663, impairs OATP1B1 transport activity [115].

Extensive work has investigated the role phosphorylation in regulating OATP function, expression and overall susceptibility to drug interactions precipitated by kinase inhibitors. Protein kinase C (PKC) activation reduces transport activity of OATP1B3 in primary human hepatocytes, although surface and total protein expression remain unchanged [116]. However, some studies have linked the signaled recycling and degradation of OATP1B1 and OATP1B3 with PKC activity. OATP1B1/3 degradation is believed to primarily occur in the lysosome [117]. After internalization with clathrin-coated pits, OATP1B1 recycling is regulated by PKC [118]. When PKC is activated, OATP1B1 remains in Rab11-positive endosomes [118]. Rab11 indicates a recycling endosome, rather than one that will be degraded. OATP1B1 activity is similarly modified by tyrosine phosphorylation. The SRC family kinase YES1 mediates tyrosine phosphorylation of OATP1B1, leading to increased uptake activity [119]. The reader is directed to recent reviews and key studies on the phosphorylation of OATPs in lieu of a more detailed discussion herein [116,120,121]. Lastly, although the overall significance remains to be determined, when ubiquitinated, OATP1B3 has been shown to also be degraded through the UPS system. This likely plays a minor role in total degradation of the protein [117] but may be relevant in pathogenesis if the UPS system is altered or targeted.

### 2.5. Multidrug Resistance Proteins (MRPs; ABCC1-9)

#### 2.5.1. Cellular Localization and Function

Multidrug resistance proteins (MRPs) have also been shown to play a role in bile acid transport, though not all of them are involved. Among the *ABCC* gene family, MRP2 (*ABCC2*), MRP3 (*ABCC3*), and MRP4 (*ABCC4*) are the primary MRPs involved in bile acid transport [122]. MRP1 (*ABCC1*), MRP5 (*ABCC5*), MRP6 (*ABCC6*) and other family members primarily transport glutathione conjugates, cyclic nucleotides, or organic anions rather than bile acids [122].

MRP2 is located at the canalicular membrane of hepatocytes (Figure 1), where it effluxes conjugated metabolites from the cell, including bilirubin conjugates [123]. MRP2 has a role similar to BSEP in canalicular excretion of bile acids, though it is a much more minor role. MRP3 is located on the basolateral membrane of hepatocytes, and it is known to mediate the efflux of conjugated bile acids and bilirubin [18]. It functions primarily as an escape route for the basolateral efflux of bile acids in times of bile acid accumulation, or when alternate export routes are required to compensate for blocked or damaged primary routes. MRP4 is believed to have dual membrane localization (apical and basolateral), with its cellular localization appearing to be cell specific. MRP4 is located in the basolateral membrane of hepatocytes and the apical membrane of renal proximal tubule cells [124].

#### 2.5.2. Regulation of MRP2 Expression and Activity

Expression of MRPs is regulated by several nuclear receptors to allow adaptive responses to xenobiotics and fluctuations in levels of bile acids [125]. Activation of CAR and PXR both induce transcription of *MRP2* and *MRP3* genes (*ABBC2* and *ABCC3*), increasing hepatocellular efflux of bilirubin conjugates, bile acids, and xenobiotic metabolites [126]. Additionally, FXR helps provide a hepatoprotective response under cholestatic conditions by increasing expression of MRP2 [126,127]. MRP2 expression is induced when FXR binds to an IR-1 FXRE element within the *MRP2* gene promoter [127]. In conditions in which FXR function is impaired, MRP4 expression is increased as an adaptive, hepatoprotective response [125]. The *MRP3* gene promoter region has recognition sites for both specificity protein 1 (SP1) and LRH-1 [18]. MRP3 expression is induced by several cytokines. The Jun amino-terminal kinase/stress-activated protein kinase (JNK/SAPK) pathway, when activated by TNF-α, leads to the induction of *MRP3* [18]. TNF-α activates JNK signaling, which then increases SP1 transcriptional activity. This promotes LRH-1-dependent promoter activation, enhancing ABCC3 transcription, representing an adaptive response to hepatic injury to prevent bile acid accumulation [18].

MRP2 undergoes post-translational regulation though a pathway dependent on the activation of liver PKCs [128]. The human ezrin protein is part of a complex that makes up radixin, which helps connect plasma membrane proteins with actin filaments to help organize cell structure [128,129]. Ezrin phosphorylation impedes the interaction between MRP2 and the actin cytoskeleton. PKCs will phosphorylate ezrin on Thr567, which, under cholestatic conditions, leads to MRP2 internalization and eventual lysosomal degradation of the transporter protein [128].

### 2.6. Bile Salt Export Pump (BSEP; ABCB11)

#### 2.6.1. Cellular Localization and Function

The bile salt export pump (BSEP) is a transport protein located on the apical membrane of hepatocytes (Figure 1) [123]. BSEP is the primary transporter responsible for canalicular export of bile acids from the hepatocyte to the biliary system [123]. Loss of BSEP leads to intracellular retention of bile acids, oxidative stress, and membrane injury [130]. Dysregulation of BSEP leads to intrahepatic cholestasis [131]. Because of the toxicity associated with bile acid accumulation, BSEP plays a central role in hepatoprotection through its transport activity. The affinity of bile acids human BSEPs is TCA > GDCA > GCA > TDCA [132].

#### 2.6.2. Regulation of BSEP Expression and Activity

BSEP expression is tightly controlled at the transcriptional level. FXR:RXR heterodimers bind to IR-1 response elements (5′-GGGACA T TGATCCT-3′) in the proximal *ABCB11* promoter [133]. This induces transcription of BSEP under conditions of elevated bile acid concentration to prevent intracellular bile acid overload. However, BSEP expression is repressed during pregnancy due to elevated levels of estrogen and progesterone metabolites, each of which antagonize FXR signaling, leading to impaired transcription of BSEP [134]. Pregnancy alters bile acid homeostasis through hormonal regulation of bile acid transporters, with BSEP being especially implicated in this process. Estradiol is able to repress FXR-mediated BSEP transcription via direct interactions between estrogen receptor α and FXR [135]. Similarly, sulfated metabolites of progesterone, like epialloprenanolone sulfate, act as FXR antagonists, reducing induction of BSEP [134].

At the protein level, after BSEP synthesis, transporter localization, stability, and activity are regulated through post-translational mechanisms. Trafficking of BSEP to the apical membrane is driven by endosomal sorting complexes required for transport III (ESCRT-III) [136]. When K63-ubiquitin linkages are formed on BSEP, ESCRT-III machinery, like charged multivesicular body protein 5 (CHMP5), targets BSEP for trafficking [136]. This may regulate the recycling of BSEP, as well as the degradation of BSEP. Rab11 endosomes may also play a role in the intracellular trafficking of BSEP [137]. Rab11-positive endosomes allow recycling of BSEP by facilitating reinsertion of the protein into the canalicular membrane [137]. This allows for more rapid maintenance of bile acid exports, as recycling prevents the time it would take to synthesize and traffic a new protein. A misfolded BSEP appears to be degraded through an endoplasmic reticulum-associated degradation (ERAD) pathway first involving ubiquitination followed by proteasomal degradation [36].

Phosphorylation of BSEP is another important post-translational modification for BSEP localization. Phosphorylation by p38 MAPK promotes trafficking of the transporter to the plasma membrane [138]. N-linked glycosylation is also required for BSEP stability, trafficking, and transport functions. Within the first extracellular loop of BSEP, there are four N-linked glycosylation sites [139].

## 3. Altered Bile Acid Transporters in Acute and Chronic Liver Diseases

### 3.1. Acute Liver Injury

#### 3.1.1. Background

Acute liver injury (ALI) is defined as the rapid onset of hepatocellular damage and presents clinically with raised levels of transaminases and an international normalized ratio (INR) score ≥ 1.5, indicating coagulopathy [24]. This condition can progress to acute liver failure, which is an often-fatal disease due to multi-organ failure [24]. The most common causes of ALI are viral infections, such as hepatitis A, B, or C, or drug-induced toxicity, such as by acetaminophen [24,140]. Drug-induced liver injury (DILI) is a condition in which exogenous compounds cause injury and death in hepatic cells [141]. DILI presentation is divided into two categories—intrinsic and idiosyncratic [142]. Intrinsic DILI is the predictable result of a dose-dependent response to a drug, while idiosyncratic DILI is spontaneous, and the underlying mechanisms causing it are not well understood [142]. Understanding the mechanisms of DILI is essential for the creation of safe and efficacious drugs, and DILI has been studied immensely. Bile acid homeostasis is believed to play an important role in DILI, as various drug metabolites may inhibit bile acid transporters [130]. Additionally, sepsis can induce ALI at an estimated frequency of 34–46% of all sepsis patients [143,144].

During the acute phase following a liver injury, multiple liver cell types are implicated in the injury repair process, aimed at protecting against liver damage and hepatocyte death. However, they may also contribute to the progression of liver disease if the underlying tissue repair mechanisms are not resolved. Kupffer cells (KCs) are the resident liver macrophages, located in the hepatic sinusoid [145]. When activated in response to liver injury, KCs will secrete chemokines and cytokines [146]. In ALI, when KCs are activated, they are responsible for the secretion of TNF-α, IL-6, and IL-1β which leads to the adaptive changes to transcriptional programs, as well as transporter response [146]. Hepatic stellate cells (HSCs) are located in the Space of Disse in the liver [147]. In response to cytokine signaling during liver injury, such as that driven by KCs, HSCs will transform from the quiescent state to the activated state [147]. In the quiescent state, HSCs will store vitamin A, but this ability is rapidly lost once HSCs become activated [147,148,149]. Once activated, HSCs will secrete collagen as a response to injury [150]. STAT3 mRNA expression has been shown to be upregulated in ALI. When STAT3 is inhibited, HSCs are able to return to a quiescent state [151].

Hepatic bile acid transporters play a critical role in the modulation of ALI disease progression and hepatic adaptation, as described in the following subsections. The cytokines released by KCs in ALI will lead to suppression of bile acid uptake, impairment of canalicular export, and the promotion of basolateral efflux transporters. This shapes the hepatocyte environment for bile acid handling during the early stages of ALI, representing an important pathway for cell-to-cell communication. Bile acids may also directly or indirectly activate HSCs. This activation of HSC proliferation was caused by an EGFR-mediated pathway [152]. However, HSC apoptosis was not induced by excess levels of the bile acids tested [152]. This study determined that NTCP was not expressed in HSCs, as fluorescent bile acids were not taken up into the cells [152]. However, another study has found NTCP expression in HSCs and observed that NTCP expression is correlated with the severity of liver fibrosis [37]. More work needs to be done to determine the role of bile acid transporters in HSCs.

The effects of several bile acids have been tested on HSCs. CA, GCDCA, and TCDCA at a concentration of 25 µM led to a 2.5–3-fold increase in HSC proliferation [152]. Plasma bile acid concentrations ≥ 25 µMare indicative of cholestasis. At 5µM, the typical physiological concentration of the bile acids tested, HSC proliferation remained unchanged [152]. Secondary unconjugated bile acids have been shown to play a stronger role in HSC activation than primary, unconjugated primary, and conjugated secondary bile acids [153]. In the LX-2 cell line, LCA had the highest ability to activate the cells [153]. Together, secondary unconjugated bile acid treatment (LCA, DCA, and UDCA) led to the activation of 20% of LX-2 cells, compared to secondary conjugated bile acids (GUDCA and GDCA) only activating around 2% of LX-2 cells [153]. Notably, taurine-conjugated bile acids were not tested in this study [153].

Finally, bile acid signaling can induce the release of cytokines and damage-associated molecular patterns (DAMPs) [154]. These cytokines and DAMPs will signal to the HSCs to activate. TGR5 expression in KCs allows signaling between the KCs and bile acids. In mice fed a diet of 1% CA, IL-1β and TNF-α mRNA levels were both increased nearly 2-fold compared to mice that were not fed CA [155].

#### 3.1.2. Hepatic Transporter Reprogramming in ALI (Broadly Defined)

Due to the sudden-onset nature of ALI, rapid, coordinated changes in bile acid transporter expression and localization must occur. This is done to prevent further damage caused by inflammation and metabolic stress. Our understanding of this process is largely centered on changes to hepatic transporters; however, the dynamic regulation of intestinal bile acid transporters has not been investigated. The changes in hepatic bile acid transporter expression seen during ALI provide an alternate route for bile acid clearance when typical pathways are disrupted during injury, while also preventing influx into the cells, helping to prevent bile acid accumulation. For instance, NTCP is downregulated at the basolateral membrane of hepatocytes in ALI [15]. A mechanistic study in a human liver-slice culture model indicates a potential role of cytokines in this process [15]. TNF-α and IL-1β suppress NTCP gene expression in human liver slices, which results in downregulation of bile acid uptake [15]. In addition to reduced expression, OATP1B1 and OATP1B3 demonstrate reduced transport activity in the presence of IL-6 and TNF-α, both of which are upregulated in ALI [16,17]. STAT3 also plays a role in the downregulation of NTCP mRNA expression [73]. Given the role of STAT3 in the promotion of HSC activation, further mechanistic work linking the STAT3 reduction in hepatocyte uptake of bile acid to the subsequent activation of HSCs by bile acid signaling in non-parenchymal cells is warranted. Overall, the downregulation of NTCP gene and protein expression is likely a protective mechanism to prevent exacerbation of ALI by bile acids but may also lead to altered signaling elsewhere.

In parallel to the downregulation of bile acid uptake transporters, dynamic changes in the expression of hepatic efflux transporters also occur during acute liver injury. TNF-α, IL-1β and IL-6 promote the reduction in MRP2 and BSEP mRNA in liver-slice cultures [15]. Conversely, MRP3 gene expression is induced by TNF-α [18], providing a compensatory route for bile acid hepatic clearance during liver injury when there is dysfunction in the typical bile acid efflux mechanisms. Because ALI is characterized by rapid onset of stress, and, eventually, death of hepatocytes, early regulation of bile acid uptake is a critical adaptive response to prevent further cellular toxicity.

#### 3.1.3. Hepatic Transporter Reprogramming in Drug-Induced Liver Injury (DILI)

Due to the cytotoxic nature of bile acids, disruption in enterohepatic circulation can prove dangerous. BSEP inhibition has been implicated in DILI, and 95% of compounds that had a steady-state concentration (C_ss_)/BSEP IC_50_ ratio greater than or equal to 0.1 were associated with liver injury [22]. When drugs additionally had a C_SS_/MRP4 IC_50_ ratio greater than or equal to 0.1, the correlation with liver injury was 100% [22]. Screening drugs for their potential to inhibit BSEPs or MRPs could be helpful in determining their safety for human use.

Acetaminophen is the most common cause of DILI [19]. In mice, acetaminophen was shown to disrupt the blood–bile barrier [19]. Eventually, the intracellular concentration of bile acids is high enough to induce hepatocyte death, leading to liver injury [19]. In mice, the inhibition of NTCP and OATP mitigates acetaminophen-induced liver injury [19]. However, further human studies are needed to validate this therapeutic approach for acetaminophen-induced liver injury. The drug bosentan, which is used to treat pulmonary arterial hypertension, induces hepatotoxicity in humans despite being assumed safe after low levels of hepatotoxicity were observed in rodent models [20]. However, in rodents, bosentan has a higher capacity to inhibit Ntcp than it does in humans, suggesting that combined inhibition of NTCP may protect against the toxic effects of BSEP inhibition [20]. Another mechanism of bile acid transport disruption in DILI is through drug interactions with FXR or FXR-mediated signaling pathways. BSEP and MRP2 mRNA expression are induced by FXR binding to sirtuin 1 (SIRT1) [23]. The drug isoniazid prevents this heterodimerization, thus lowering the expression of BSEP and MRP2, allowing bile acid accumulation within the hepatocyte [23]. For DILI induced by chlorpromazine (CPZ), oxidative stress seems to play a role in the dynamic regulation of bile acid transporter expression [21]. NTCP expression and activity decreased, BSEP and MDR3 expression decreased, and MDR4 expression increased after exposure to CPZ [21].

### 3.2. Cholestasis

#### 3.2.1. Background

Cholestasis results in high intracellular bile acid concentrations and is a consequence of altered bile flow stemming either from physical obstruction of bile flow or impaired hepatic biliary secretion of bile acids [156]. There are several different types of cholestasis, each varying in their etiology. Progressive familial intrahepatic cholestasis (PFIC) is driven by genetic mutations in hepatocellular transport systems [157]. Intrahepatic cholestasis of pregnancy (ICP) occurs primarily during the second and third trimesters of pregnancy, though its causes are multifactorial and not well understood [158]. Extrahepatic cholestasis is caused by obstructions in the biliary system outside of the liver, such as gallstones or tumors [159].

Due to the impairment of hepatocellular bile acid export in cholestasis, hepatocytes are at risk of accumulating intracellular bile acids, leading to toxicity and liver injury. Despite the diversity in the subtypes of cholestasis, bile acid transporters undergo several changes in expression and activity in each [160]. In addition, variations in bile acid transporters during cholestasis may be either causative of the condition, or a result of an adaptive change to the new bile acid environment. These dynamic effects on bile acid transporter expression are outlined in the following disease-specific subsections.

#### 3.2.2. Alterations to Bile Acid Transporters in PFIC

PFIC has three subtypes. In PFIC 1, also known as Byler disease, *ATP8B1* is mutated [26]. ATPase phospholipid transporting 8B1 (*ATP8B1*) encodes a phospholipid flippase that transports phospholipids from the extracellular environment into the cell [26]. This is believed to render the cellular membrane unstable, leading to reduced cellular ability to transport bile acids out of the cell; however, the exact mechanism of bile acid accumulation in PFIC 1 is not known [26,161]. Like other cholestatic conditions, FXR expression and activity are reduced in PFIC 1 [161,162]. PKC zeta is implicated in this reduction in FXR activity through increased phosphorylation of the nuclear receptor during disease [163]. This impairs feedback inhibition of bile acid synthesis [162]. PFIC 2, also known as BSEP disease, is caused by mutations in *ABCB11* [26]. The most common mutations responsible for PFIC II are E297G, D482G, and N591S [164]. This disease is explicitly caused by deficient BSEP, with specific mutations proving to be more severe than others. Patients with p.D482G or p.E297G *BSEP* mutations, for example, are less likely to progress to hepatocellular carcinoma (HCC) than patients with nonfunctional *BSEP* mutations (7% vs. 34%) [27]. Furthermore, NTCP protein has been shown to be downregulated along with OATP1B3 and OATP1B1 mRNA levels in PFIC 2 [25]. These changes likely represent an adaptive hepatoprotective response to elevated bile acid concentrations, where the suppression of uptake transporters limits further bile acid accumulation [164]. Although speculative, this may be secondary to increased bile acid signaling due to increased cellular bile acid concentrations. PFIC 3, also known as multidrug resistance 3 (MDR3) disease, is caused by mutations in ATP-binding cassette subfamily B member 4 (*ABCB4*) [26]. In PFIC 3, NTCP protein is also downregulated [24]. OATP1B3 and OATP1B1 mRNA levels are also reduced in PFIC 3, although not to the same level as in PFIC 2 [25]. In each PFIC, MRP3 mRNA and protein levels are unchanged. However, in all types of PFIC, MRP4 expression is upregulated at both the mRNA and protein levels, suggesting that MRP4 may act as an escape route for bile acids under cholestatic conditions [25].

#### 3.2.3. Alterations to Bile Acid Transporters in ICP

ICP causality remains poorly understood but is thought to be influenced by a combination of hormonal, environmental, and genetic factors [165]. Several bile acid transporters have been studied in relation to this condition. Like in PFIC, ABCB4 has been implicated in ICP. Heterozygous ABCB4 mutations are associated with ICP [29]. These mutations limit the ability of MDR3 to transport phosphatidylcholine, preventing protection against bile acid-induced cytotoxicity, and leading to the presentation of cholestasis.

Heterozygous mutations in BSEP are also associated with ICP, rather than the homozygous mutations seen in relation to PFIC 2 [29]. However, BSEP expression is repressed during pregnancy due to elevated levels of estrogen and progesterone metabolites, each of which antagonize FXR signaling, leading to impaired transcription of BSEP [134]. Pregnancy alters bile acid homeostasis through hormonal regulation of bile acid transporters, with BSEP being especially implicated in this process. Estradiol is able to repress FXR-mediated BSEP transcription via direct interactions between estrogen receptor α and FXR [135]. This leads to reductions in BSEP transport capacity in patients with ICP [135]. Progesterone further impairs BSEP regulation during pregnancy [134]. Sulfated metabolites of progesterone, like epialloprenanolone sulfate, act as FXR antagonists, reducing the induction of BSEP [134]. Treatment with ursodeoxycholic acid (UDCA) is the primary treatment of ICP and is associated with pruritis [166,167,168]. Many mechanisms for its apparent anticholestatic efficacy have been suggested, including the stimulation of biliary efflux of bile acids and kinase-mediated upregulation of BSEP and MRP2 membrane expression [167,169,170].

Induction of MRP2 can also occur through FXR activation [127]. Interestingly, unlike in other forms of cholestasis, hyperbilirubinemia is not commonly observed in ICP [171]. This observation suggests preserved hepatic clearance of bilirubin glucuronides by MRP2 in pregnancy [172,173], possibly synergistically with the upregulation of UDP-glucuronosyltransferase (UGT) 1A1 by pregnancy hormones [174,175]. Supporting the protective role of MRP2 against hyperbilirubinemia in ICP, a single polymorphism of MRP2 frequently observed in South American populations was associated with ICP. The same effects were not seen in Caucasian populations, highlighting the multifaceted causes of this condition [176,177]. In addition to mutations in individual transporters, genetic alterations in upstream regulatory pathways further contribute to ICP pathogenesis. The M173T mutation in FXR was significantly associated with ICP in Caucasian women [178]. This mutation affects the DNA-binding domain of FXR, making it unable to properly activate BSEP or MDR3 (96). M1V and –1 G > T mutations in FXR impact the translation efficiency of FXR, preventing protein from being formed as efficiently [178].

Extrahepatic changes to bile acid disposition have been shown to occur in ICP. For instance, in the placenta, the expression of organic anion transporting polypeptides may be impacted. Placental SLCO3A1 was significantly downregulated in patients with ICP compared to those without ICP [28]. OATP1A2 and OATP1B3 were shown to be changed during ICP; however, there is only a small cohort from which data was collected [28]. UDCA treatment in ICP has been suggested to have a feto-protective role by inducing the placental gene expression of the breast cancer resistance transporter (BCRP) involved in bile acid efflux [168]. Further work is needed to corroborate these findings in larger cohorts as ICP is a well-established driver of adverse pregnancy outcomes, such as preterm birth and increased risk of stillbirth [179].

#### 3.2.4. Alterations in Bile Acid Transporters in Extrahepatic Cholestasis

Extrahepatic causes of cholestasis lead to obstruction of bile acid flow, leading to secondary accumulation of bile acids within the hepatocyte [180]. As a response to this dysregulation in the bile acid environment, several transporters undergo compensatory changes in their expression levels, influencing their relative importance in maintaining bile acid homeostasis under cholestatic conditions.

In obstructive cholestasis, reduced enterohepatic recycling and intestinal uptake of bile acids by ASBT protects the liver against injury [55]. In mice, knockout of Asbt was able to effectively reduce bile salt pool size, as well as prevent damage to the liver [55]. In humans with obstructive cholestasis, ASBT is downregulated at the mRNA level by 4-fold [181]. The ASBT inhibitor linerixibat is FDA approved to treat pruritis caused by primary biliary cholangitis (PBC) [182]. Despite its efficacy, gastrointestinal side effects are observed in patients who receive linerixibat compared to a placebo [182]. Additionally, in trials, severe side effects were seen in 12% of treated patients versus only 3% of placebo patients [182]. In the liver, the bile acid uptake transporter NTCP is reduced at both the mRNA and protein levels to prevent overload of bile acid uptake into the hepatocyte. MRP2, BSEP, and MDR1 mRNA levels remained stable compared to healthy patients [18]. However, MRP3 mRNA and protein levels were significantly increased [18]. This represents an alternate route for the efflux of bile acids compared to healthy patients. The increase in MRP3 mRNA levels occurs due to TNF-α signaling following activation by JNK/SAPK and SP1 [18].

Similarly, OATP3A1 mRNA and protein levels are induced in obstructive cholestasis [183]. TNF-α induces OATP3A1 through the binding of NF-κB, p65, and SP1 to the OATP3A1 gene promoter region, thereby inducing its transcription [183]. This provides another compensatory route for bile acid excretion from hepatocytes. Thus, TNF-α appears to play a dual role, contributing to liver injury while simultaneously promoting adaptive transporter expression to limit intracellular bile acid accumulation.

In addition to ASBT inhibition, a number of agonists for peroxisome proliferator-activated receptors (PPARs) have been clinically evaluated for the treatment of primary biliary cholangitis (PBC). They upregulate MRP3 expression to promote bile acid efflux from the liver, inhibiting de novo bile acid synthesis while upregulating BA detoxification mechanisms [184]. In 2024, two PPAR agonists, elafibranor [185] and seladelpar [186], were approved in the United States for the treatment of PBC in patients who are non-responsive to UDCA.

### 3.3. Metabolic Dysfunction-Associated Steatotic Liver Disease (MASLD) and Steatohepatitis (MASH)

#### 3.3.1. Background

Metabolic dysfunction-associated steatotic liver disease (MASLD) is a progressive liver condition often accompanied by metabolic disorders, including obesity, and is attributed to excessive accumulation of triglycerides (steatosis) in the liver [187]. Advanced stages of MASLD are referred to as metabolic dysfunction-associated steatohepatitis (MASH). In MASH, hepatic steatosis is accompanied by lipotoxicity, ER and oxidative stress, along with mitochondrial dysfunction. In addition, the activation of KCs and HSCs increases liver cytokine concentrations, promoting tissue remodeling, fibrogenesis, and hepatocyte ballooning [187]. Chronic MASLD/MASH inevitably progresses to liver fibrosis and hepatocellular carcinoma (HCC) [188], which are further discussed later in this review.

The etiology of MASLD/MASH is complex and influenced by underlying metabolic comorbidities. In addition, individuals may be predisposed to MASLD/MASH by genetic and environmental factors including diet [189]. The pathogenesis of MASLD/MASH therefore is described as following the “multiple-hit” hypothesis, as several causes influence the development of MASH from MASLD [190]. Disruptions in bile acid metabolism and homeostasis are closely associated with worsening MASLD [191]. As transcriptional regulators of lipid metabolism and cholesterol homeostasis, bile acids are recognized as potential effectors of MASLD/MASH progression rather than passive metabolic byproducts of the condition. As such, understanding changes in the bile acid metabolome and the transporters mediating their cellular disposition is likely to provide important insight into the pathogenesis of MASLD/MASH.

#### 3.3.2. Changes to the Bile Acid Metabolome in MASLD/MASH

In the progression from metabolically healthy to MASLD, and MASLD to MASH, the bile acid pool composition undergoes many changes. Specific species of bile acids may increase in advanced disease states, such as DCA [56,191,192]. Additionally, oxidized bile acids, including 7-keto-DCA and 7-keto-LCA, are associated with increased MASH severity [193]. Overall, though, the total amount of serum bile acids is increased for patients with MASH [189]. This same study also saw increased expression of CYP7A1, the enzyme responsible for bile acid synthesis [189]. The secondary bile acid, 3-succinyl cholic acid (3-sucCA), was particularly lowered in patients with MASLD [194]. Lower levels of 3-sucCA were also correlated with higher levels of the liver enzymes alanine aminotransferase (ALT) and aspartate aminotransferase (AST), indicators of liver injury [194]. An LC-MS analysis of 65 bile acids in patients with MASLD proposed that six bile acid metabolites may be able to be used as a diagnostic tool for the condition [195]. Glycohenodeoxycholate-3-sulfate, glycoursodeoxycholate-3-sulfate, chenodeoxycholate-3-sulfate, norcholic acid, and taurochenodeoxycholic acid were each significantly increased, while hyodeoxycholic acid was significantly decreased in a manner that was correlated to liver injury severity [195].

However, the mechanistic effects of individual bile acids on disease pathogenesis remain understudied. For instance, it is unclear how changes to specific bile acids alter transcriptional programs, and whether these changes are a direct or indirect consequence of the observed increases or decreases in the effected bile acid metabolites. Additionally, a critical assumption of these findings is that hepatic tissue concentrations mirror changes in the plasma. This is unlikely to be true given the high extraction of bile acids into hepatocytes and capacity for biotransformation in the liver. Last, much of the bile acid metabolomics work on humans in the literature is from untargeted metabolomic screens and do not provide absolute, quantitative metrics (e.g., nM concentrations) for the changes observed in plasma. This may over- or under-estimate the biological significance of specific bile acid metabolites in circulation. Thus, more work remains to be done in this area to determine the diagnostic potential and causative role of bile acid in human MASLD or MASH, but there is ample evidence in the literature to support the premise that bile acid composition may influence disease progression.

#### 3.3.3. Changes in Bile Acid Transporters in MASLD/MASH

MASLD and MASH are associated with significant changes to hepatocellular bile acid transporter expression and localization. In response to lipid overload and bile acid-induced toxicity, bile acid uptake and efflux pathways are reprogrammed. As the liver progresses from steatosis to steatohepatitis, these adaptative responses prove critical. Sinusoidal bile acid uptake is increased in MASLD and MASH, though changes occur dynamically during the progression from MASLD to MASH. Therefore, depending on the disease state, the relative changes in the expression of individual bile acid transporters may lead to variability in the extent and direction of change to cellular bile acid concentrations and subsequent signaling potential. For instance, compared to healthy individuals, patients with MASLD show upregulated levels of NTCP [30]. During the progression from MASLD to MASH, liver expression of NTCP appears to decrease but remains elevated when compared to healthy human liver controls [31]. In the later stage, downregulation of sinusoidal uptake likely represents a compensatory mechanism, as the liver attempts to respond and mitigate the increased concentrations of bile acids in the hepatocyte by reducing hepatocyte extraction of enterohepatically recycled bile acids. In addition to NTCP, OATP1B3 mRNA and protein expression are increased in MASLD [31]. Due to the upregulation, NTCP inhibitors have been used therapeutically, such as Myrcludex B (MyrB), an NTCP inhibitor originally made for the treatment of HBV and HDV [196]. MyrB has also been shown to promote the release of glucagon-like peptide 1 (GLP-1), which helps patients with obesity [197]. Another NTCP inhibitor, hepalatide, also prevents the progression of MASLD in mice [198]. However, most NTCP inhibitors have been studied in the context of viral infection, so more work remains to be done to determine their efficacy in progressive liver diseases.

In parallel to sinusoidal changes, canalicular efflux of bile acids is downregulated in MASLD and MASH. MRP2 function was shown to be reduced in patients with MASH [33]. A decrease in BSEP expression was associated with worsening MASLD activity scores [32]. As a result of this decrease in canalicular efflux, sinusoidal bile acid efflux is often induced as an escape route for bile acids. OSTα/β expression is also increased in patients with MASH [35]. MRP3 is also induced [34]. While these changes on both the sinusoidal and canicular membrane are in part transcriptionally driven, it is unknown how the protein function is altered in MASLD/MASH and there likely are critical mechanisms driven by post-translational modification and/or protein–protein interactions that influence transporter function.

In contrast to the changes in the liver, far less is known about the regulation of intestinal ASBT and OSTα/β in humans with MASLD/MASH. However, given the role of bile acid flux and signaling in mediating metabolic perturbation in MASLD/MASH, the inhibition of intestinal bile acid uptake has been investigated as a therapeutic approach. In a phase 1 clinical trial, the ASBT inhibitor volixibat showed improved LDL cholesterol levels in a cohort of participants with obesity but failed in a phase 2 clinical trial focused on MASH [199,200]. Additionally, the vast majority of participants enrolled in the trials experienced treatment-emergent adverse events, including diarrhea and nausea [200].

Collectively, bile acid transport in MASLD/MASH is remodeled as an adaptive response to liver injury. The hepatocytes shift from increased bile acid uptake in the early stages of disease to suppression of NTCP and OATPs as liver impairment progresses [201]. Rather than an adaptive response though, BSEP activity is reduced in the hepatocyte [202]. This limits canalicular efflux of bile acids, driving disease progression as bile acids induce intracellular stress [202]. Hepatocytes in patients with MASLD and MASH begin to balloon as a result of damage from excess lipid deposits [203] and HSCs activate continuing disease progression [203]. Finally, NOTCH signaling in MASLD and MASH also pushes hepatocyte progenitor cells toward cholangiocyte differentiation [204]. Cholangiocytes are more resistant to lipotoxicity than hepatocytes, which may be why their differentiation is favored in a disease-state environment [204].

### 3.4. Liver Fibrosis and Cirrhosis

#### 3.4.1. Background

Liver fibrosis occurs when chronic damage occurs in the liver, and excess extracellular matrix proteins are deposited into the liver [205]. The activation of HSCs is a hallmark of liver fibrosis. Prolonged liver impairment and injury occurring in conjunction with liver fibrosis promotes the progression to liver cirrhosis, defined as diffuse scarring in the liver [205]. Bile acid homeostasis is disrupted significantly in both conditions, leading to further changes in bile acid transporter expression than the changes described in the preceding section on MASLD/MASH.

KCs and HSCs each play a key role in driving liver fibrosis and its progression to cirrhosis. When HSCs transition from the quiescent state to the activated state, their production of type I and type III collagen is increased [206]. This is deposited into the extracellular matrix, leading to the progression of fibrosis. HSC activation is induced by cytokines released from KCs [207]. However, early in the signaling cascade triggered by hepatic cytokines and preceding stellate cell activation, cytokines acutely regulate a number of genes involved in vitamin A metabolism and mobilization from lipid droplet stores [148,149]. Bile acids have also been shown to have the ability to indirectly activate HSCs by activating EGFR [152]. Bile acids induced the phosphorylation of EGFR through ROS-mediated interaction with EGFR [152]. This initiates a PKC-ERK-p70S6K signaling pathway that ultimately leads to the activation of HSCs [152]. FXR inhibits HSC activation as well, thereby preventing liver fibrosis, by SHP induction [208]. In liver fibrosis, bile acid plasma concentrations are generally increased, especially CDCA, which may promote proliferation of HSCs and contribute to their activation. Conversely, KCs express the bile acid membrane receptor, TGR5. This suggests a direct effect of bile acids on KCs [209]. In vitro inhibition of TGR5 blocked the activation of HSCs [209]. Thus, it appears that bile acid may both promote and prevent fibrogenesis in the liver. This may support their general function as a mediator in wound-repair and tissue remodeling processes after liver injury. However, further in vitro mechanistic work is needed to elucidate and translate their importance to liver disease pathogenesis.

#### 3.4.2. Changes in Bile Acid Transporters in Liver Fibrosis and Cirrhosis

In livers from patients with cirrhosis, transporter protein expression was changed depending on the identity of the transporter or the cause of cirrhosis. In patients with alcohol-induced cirrhosis, each of the liver transporters had reduced expression except MRP3, which had increased expression. In hepatitis C-induced cirrhosis, however, BSEP, MRP2, NTCP, and OATP1B3 each had reduced expression, while the expression of MRP3 and OATP1B1 was unchanged.

One study evaluating mRNA expression of drug uptake and efflux transporters reported increased expression of MRP4, OATP1B1, and OATP1B3, suggesting a compensatory response to cirrhosis [210]. In contrast, BSEP and OATP2B1 mRNA expression were reduced in liver cirrhosis [211].

Loss of function of bile acid transporters also plays an important role in liver fibrosis and cirrhosis pathogenesis. In a case study of a child with a loss-of-function OSTα mutation, early onset liver fibrosis was developed [212]. Contrastingly, Bsep knockout mice were protected from toxin-induced liver fibrosis development [213]. pJNK signaling in Bsep knockout mice resulted in reduced activation of HSCs, thus preventing fibrosis [213]. The bile acid pool composition was changed to contain a higher ratio of tetrahydroxylated bile acids, which drove this attenuation of HSC activation as a result of increased p62 and Nrf2 gene expression, as well as decreased AP-1 activity [213].

While HSCs have not traditionally been considered to express bile acid uptake transporters, a recent study showed expression of NTCP in primary HSCs [37]. This study further demonstrated higher expression of NTCP in HSCs as liver fibrosis advanced, as NTCP expression was three-fold higher in the F3/4 stage of liver fibrosis compared to the F0 phase of disease [37]. In addition to increased expression of NTCP, polymorphisms play a role in the severity of liver fibrosis. The rs4646287 NTCP polymorphism is specifically associated with the development of liver fibrosis after HBV infection [37].

### 3.5. Hepatocellular Carcinoma

#### 3.5.1. Background

Hepatocellular carcinoma (HCC) is a primary liver tumor and occurs frequently in patients who have liver cirrhosis [214]. Like many other conditions, predisposition to HCC is multifactorial, and often involves a combination of genetic and environmental risk factors. Central to HCC is the chronic disruption of bile acid homeostasis, as well as a shift away from adaptive transporter remodeling seen in earlier stages of progressive liver diseases. Specifically, HCC is characterized by loss of FXR [215], resulting in suppression of both bile acid uptake (NTCP) and efflux (BSEP) pathways and driving accumulation of bile acids both in the liver and in circulation. In turn, the toxic effects of hepatic accumulation of bile acids can mediate FXR-independent signaling, thereby promoting major changes to the liver environment, tumor cell proliferation, and carcinogenesis [216]. Notably, the regulation of bile acid homeostasis is a significant target of HCC treatments, as recently reviewed by Wang et al. [217]. Many of these treatments alter signaling pathways implicated in the dynamic regulation of bile acid transporters. However, clinical evidence is needed to determine if transporter expression and bile acid transporter activity change in patients throughout their treatment regimens.

#### 3.5.2. Changes in Bile Acid Signaling and Metabolome in HCC

Bile acids and their receptors are severely impacted in HCC. In mice, Fxrknockout leads to the development of HCC. In one study, all Fxr knockout mice developed liver tumors by 15 months of age, while none of the wild-type mice at the same age developed tumors [218]. In patients with HCC, FXR mRNA expression was found to be lower in tumor tissue compared to peritumoral tissue and nearly 4.5-fold lower than the mRNA expression measured in normal tissue obtained from patients without HCC (hemangiomas) [215]. This change was seen in FXR protein expression as well [215]. Additionally, patients with lower FXR expression in HCC have reduced overall survival than patients with higher FXR expression [215]. The FXR agonist obeticholic acid (OCA), has been shown to suppress HCC [219]; however, its use is not approved due to the risk of severe liver injury. This supports a hepatoprotective role of FXR, though work remains to be done to make therapeutics safe for use in patients. TGR5 hypermethylation has also been implicated in worse HCC patient survival, though levels of TGR5 protein expression remain the same between healthy patients and those with HCC [220]. This suggests that epigenetic modulation of TGR5, rather than TGR5 degradation or transcription, is altered in HCC.

Patients with HCC see significantly higher concentrations of serum bile acids which may influence hepatic bile acid signaling. TCA in particular has been shown to increase more than 450-fold in the serum of participants with HCC compared to healthy participants [221]. Notably, however, these changes reflect the severity of underlying liver dysfunction, rather than serving as HCC-specific metabolic reprogramming. Additionally, differences in bile acid concentrations had only a limited ability to determine liver cirrhosis from HCC [221]. While it is known that bile acids play a role in HCC pathogenesis, little is known about the complex mechanisms driving disease progression. Previously, CDCA signaling was shown to have transcriptional consequences on HCC through the induction of the transcription factor Snail. Snail induction by CDCA represses transcription of the tumor-suppressor gene e-cadherin. This promotes cancer cell motility, facilitating the spread of HCC. DCA induces DNA damage in HSCs through the p53/p21 pathway and leads to G0/G1 cell cycle arrest and cellular senescence. Senescent HSCs increase secretion of IL-8 and TGF-β. These cytokines in turn activate ERK and Smad signaling pathways, which allow tumor cell migration in HCC, promoting progression of the disease [222].

#### 3.5.3. Changes to Bile Acid Transporters in HCC

Downregulated *NTCP* expression has been consistently associated with worsened symptoms and outcomes of HCC [38,39]. In the hepatocellular carcinoma cell line, HepG2, H3K27 acetylation near the *NTCP* promoter is blocked compared to non-cancerous primary human hepatocytes, suggesting that HCC transcriptional changes promote NTCP repression [223]. Levels of NTCP expression may also be impacted by the cell cycle, which is strongly disrupted in HCC. NTCP ectopic expression in HepG2 and Huh7 cells lead to cell cycle arrest in the G0/G1 phase, revealing that NTCP activity or bile acid substrates may play a role as a tumor suppressor [38]. OATP1B1, OATP1B3, and OATP2B1 are all downregulated in HCC. A comparison of survival outcomes in HCC patients revealed that lower expression of *SLCO1B1* and *SLCO2B1* is associated with overall reduced survival, while no consistent relationship was observed for *SLCO1B3* [40]. Lower *SLCO1B3* gene expression was not correlated to shorter survival, despite its downregulation in HCC [40].

On the canicular membrane, *BSEP* is downregulated and associated with lower survival in HCC. This downregulation of BSEP is believed to be caused by an alteration in FXR isoforms [41]. In humans, *BSEP* expression is induced more by the FXR-α2isoform than the FXR-α1isoform [41]. Chronic exposure to IL-6 and TNF-α increases the FXR-α1/FXR-α2 ratio, leading to decreases in BSEP expression [41]. IL-6 and TNF-α are both increased in HCC [41]. Hypomethylation in BSEP (*ABCB11*) promoter region was also associated with HCC when compared to normal liver tissue [224].

Conversely, MRP4 is upregulated in HCC, which is associated with poor patient prognosis [42]. Similarly, rat Mrp1 mRNA expression was increased in an HCC rat model [225]. The *Mrp1*/*Gapdh* ratio in tumor cells from rats was twice as high when compared to non-tumor liver cells [225].

## 4. Conclusions

Bile acid transporter remodeling in liver diseases provides an adaptive response to several different pathologies. Coordinated changes in uptake and efflux pathways limit bile acid accumulation in hepatocytes, primarily through suppression of bile acid uptake and enhancement of alternate efflux mechanisms. Restoring BA transporter function represents a tractable therapeutic strategy. For instance, given that the expression profiles of bile acid transporters are largely enriched in the liver, drugs targeting them are likely to have minimal off-target effects in extrahepatic tissues, especially those mediated by perturbed and/or extrahepatic disposition of bile acids. Transporter modulators also overcome therapeutic challenges of targeting bile acid synthesis enzymes, which have indispensable roles in the catabolism of plasma cholesterol [226], and FXR agonists, which have been associated in humans with development of pro-atherogenic lipid profiles [227].

By understanding how bile acid transporters within the enterohepatic circulation are regulated, critical insight into disease pathogenesis can be gained. Although significant progress has been made in understanding bile acid regulation at the transcriptional to post-translational levels, much work remains to be done. Critical gaps explaining how phosphorylation, glycosylation, ubiquitination, and protein–protein interactions regulate transporter stability, trafficking, and function remain. How these post-translational modifications specifically impact liver disease progression also remains to be elucidated. Similarly, while it is well established that the gut microbiome is altered in all of the liver diseases described, their direct and indirect effects on bile acid transporter activity and expression are underappreciated. More mechanistic work is needed to directly link specific microbial species or secondary metabolites to the transcriptional and post-translational mechanisms described herein.

This review has left out commentary on changes to the liver architecture [228,229], zonal expression patterns of bile acid transporters [47,230,231] and their regulating proteins [230,232] in normal physiology and in liver diseases. These changes likely contribute significantly to both hepatic extraction, metabolism, signaling potential, and biliary clearance of bile acids from the liver. For instance, recent three-dimensional human liver reconstructions and volumetric determinations have shown how liver cirrhosis altered bile duct organization and branching [229]. In addition, ischemia–reperfusion injuries also have multifaceted effects on liver architecture and signaling pathways (e.g., cytokines) and thus bile acid signaling and transporter expression. Further work characterizing the structural dynamics and remodeling of the liver may elucidate additional mechanisms driving changes to bile acid transporters in liver diseases of a range of etiologies.

Although bile acids have long been proposed as targets for acute and chronic liver disease, clinical translation has been difficult for several reasons. A better understanding of the changes in bile acid pool composition is needed, as well as an understanding of the impact specific disease states have on this pool composition. Therefore, it is essential to further define the relationship and co-regulation of bile acid transporters and corresponding regulatory proteins.

## Figures and Tables

**Table 1:** Summary of bile acid transporter changes in liver disease.

Liver Disease	Direction of Change	Type of Change	Relevant References
Acute Liver Injury (broadly defined)			
Uptake Transporters			
NTCP	↓	Rapid	[13,14,15]
OATP1B1	↓	Rapid, dynamic	[16,17]
OATP1B3	↓	Rapid, dynamic	[16,17]
Efflux Transporters			
BSEP	↓	Dynamic	[15]
MRP2	↓	Dynamic	[15]
MRP3	↑	Compensatory	[18]
Drug-Induced Liver Injury (DILI)			
Uptake Transporters			
NTCP	↓	Adaptive	[19,20,21]
OATP1B1	↓	Inhibition	[19]
OATP1B3	↓	Inhibition	[19]
Efflux Transporters			
BSEP	↓	Direct inhibition	[21,22,23]
MRP2	↓	Transcriptional repression	[23]
MRP4	↓	Direct inhibition	[21,22]
Cholestasis			
PFIC			
Uptake Transporters			
NTCP (SLC10A1)	↓	Adaptive	[24,25]
OATP1B1	↓	Transcriptional repression	[25]
OATP1B3	↓	Transcriptional repression	[25]
Efflux Transporters			
BSEP	↓	Causative genetic deficiency	[26,27]
MRP4	↑	Compensatory	[25]
ICP			
Uptake Transporters			
OATP1B3	↓ (placental)		[28]
Efflux Transporters			
BSEP	↓	Genetic Variation	[29]
Obstructive Cholestasis			
Uptake Transporters			
NTCP (SLC10A1)	↓	Adaptive	[18]
Efflux Transporters			
MRP3	↑	Compensatory	[18]
MASLD/MASH			
Uptake Transporters			
NTCP (SLC10A1)	↑ (MASLD); ↓ (MASH)	Adaptive	[30,31]
OATP1B1	↑	Adaptive	[31]
OATP1B3	↓	Transcriptional Repression	[31]
Efflux Transporters			
BSEP	↓	Maladaptive	[32]
MRP2	↓	Impairment	[33]
MRP3	↑	Compensatory	[34]
OST alpha beta	↑	Compensatory	[35]
Liver Fibrosis/Cirrhosis			
Uptake Transporters			
NTCP	↑ (HSC expression); ↓ (alcohol-induced cirrhosis)	Maladaptive	[36,37]
OATP1B1	↓	Impairment	[36]
OATP1B3	↓	Impairment	[36]
*Efflux Transporters*			
BSEP	↓	Maladaptive	[36]
MRP2	↓	Impairment	[36]
Hepatocellular Carcinoma			
Uptake Transporters			
NTCP (SLC10A1)	↓	Transcriptional Repression	[38,39]
OATP1B1	↓	Transcriptional Repression	[40]
Efflux Transporters			
BSEP	↓	Maladaptive	[41]
MRP4	↑	Compensatory	[42]

This table summarizes the effect of liver disease conditions on EHC transporters. Down arrows reflect transporter downregulation, while up arrows reflect transporter upregulation.

## Data Availability

No new data were created or presented in this study.

## Abbreviations

The following abbreviations are used in this manuscript:

Abbreviation	Definition
ABC	ATP-binding cassette
ALI	Acute liver injury
ALT	Alanine aminotransferase
AP	Activator protein
ASBT	Apical sodium-dependent bile acid transporter
ATF	Activating transcription factor
ATP8B1	ATPase phospholipid transporting 8B1
BSEP	Bile salt export pump
CA	Cholic acid
CAR	Constitutive androstane receptor
CDCA	Chenodeoxycholic acid
CDX	Caudal-type homeobox
CHMP	Charged multivesicular body protein
CPZ	Chlorpromazine
CYP	Cytochrome P450
DAMP	Damage-associated molecular pattern
DCA	Deoxycholic acid
EGFR	Epithelial growth factor receptor
ER	Endoplasmic reticulum
ERAD	Endoplasmic reticulum-associated degradation
ERK	Extracellular signal regulated kinase
ESCRT	Endosomal sorting complexes required for transport
FGF	Fibroblast growth factor
FGFR	Fibroblast growth factor receptor
FXR	Farnesoid X receptor
FXRE	Farnesoid X receptor response element
GCA	Glycocholic acid
GCDCA	Glycochenodeoxycholic acid
GCDCA-S	Sulfated glycochenodeoxycholic acid
GR	Glucocorticoid receptor
HBV	Hepatitis B virus
HCC	Hepatocellular carcinoma
HDV	Hepatitis D virus
HNF1α	Hepatocyte nuclear factor 1 homeobox A
HNF4α	Hepatocyte nuclear factor 4 α
HSC	Hepatic stellate cell
IBABP	Ileal bile acid-binding protein
ICP	Intrahepatic cholestasis of pregnancy
IL	Interleukin
INR	International normalized ratio
IR	Inverted repeat
JNK	c-Jun N-terminal kinase
JNK/SAPK	Jun amino-terminal kinase/stress activated protein kinase
KC	Kupffer cells
LCA	Lithocholic acid
LDL	Low-density lipoprotein
LRH	Liver receptor homolog
LXR	Liver X receptor
MAPK	Mitogen-activated protein kinase
MASH	Metabolic-associated steatohepatitis
MASLD	Metabolic dysfunction-associated steatotic liver disease
MRP	Multidrug resistance protein
MyrB	Myrcludex B
nt	Nucleotide
NTCP	Na+/taurocholate cotransporting peptide
OATP	Organic anion transporting polypeptide
OCA	Obeticholic acid
OSTα/β	Organic solute transporter α/β
PBAM	Primary bile acid malabsorption
PBC	Primary biliary cholangitis
PFIC	Progressive familial intrahepatic cholestasis
PKC	Protein kinase c
PPARα	Peroxisome proliferator-activated receptor α
PTM	Post translational modification
PXR	Pregnane X Receptor
RAR	Retinoic acid receptor
RNA	Ribonucleic acid
ROS	Reactive oxygen species
RXR	Retinoid X receptor
SHP	Small heterodimer partner
SIRT1	Situin 1
SLC	Solute carrier
SLCO	Solute carrier organic anion transporter
SP	Specificity protein
SREBP	Sterol regulatory binding element protein
STAT	Signal transducer and activator of transcription
TCA	Taurocholic acid
TCDCA	Taurochenodeoxycholic acid
TGR	Takeda G-protein coupled receptor
TNF	Tumor necrosis factor
UDCA	Ursodeoxycholic acid
YES	Yamaguchi sarcoma viral oncogene homolog
